# Plastic expression of heterochrony quantitative trait loci (*h*QTLs) for leaf growth in the common bean (*Phaseolus vulgaris*)

**DOI:** 10.1111/nph.13386

**Published:** 2015-03-27

**Authors:** Libo Jiang, Jose A. Clavijo, Lidan Sun, Xuli Zhu, Mehul S. Bhakta, Salvador A. Gezan, Melissa Carvalho, C. Eduardo Vallejos, Rongling Wu

**Affiliations:** ^1^ Center for Computational Biology College of Biological Sciences and Technology Beijing Forestry University Beijing 100083 China; ^2^ Department of Horticultural Sciences University of Florida Gainesville FL 32611 USA; ^3^ Beijing Key Laboratory of Ornamental Plants Germplasm Innovation & Molecular Breeding National Engineering Research Center for Floriculture Beijing Laboratory of Urban and Rural Ecological Environment and College of Landscape Architecture Beijing Forestry University Beijing 100083 China; ^4^ School of Forest Resources and Conservation University of Florida Gainesville FL 32611 USA; ^5^ Center for Statistical Genetics The Pennsylvania State University Hershey PA 17033 USA

**Keywords:** common bean (*Phaseolus vulgaris*), functional mapping, heterochrony, heterochrony quantitative trait loci (*h*QTLs), quantitative trait loci (QTLs)

## Abstract

- Heterochrony, that is, evolutionary changes in the relative timing of developmental events and processes, has emerged as a key concept that links evolution and development. Genes associated with heterochrony encode molecular components of developmental timing mechanisms. However, our understanding of how heterochrony genes alter the expression of heterochrony in response to environmental changes remains very limited.
- We applied functional mapping to find quantitative trait loci (QTLs) responsible for growth trajectories of leaf area and leaf mass in the common bean (*Phaseolus vulgaris*) grown in two contrasting environments.
- We identified three major QTLs pleiotropically expressed under the two environments. Further characterization of the temporal pattern of these QTLs indicates that they are heterochrony QTLs (*h*QTLs) in terms of their role in influencing four heterochronic parameters: the timing of the inflection point, the timing of maximum acceleration and deceleration, and the duration of linear growth. The pattern of gene action by the *h*QTLs on each parameter was unique, being environmentally dependent and varying between two allometrically related leaf growth traits.
- These results provide new insights into the complexity of genetic mechanisms that control trait formation in plants and provide novel findings that will be of use in studying the evolutionary trends.

## Introduction

It has been recognized that much of morphological diversity may have resulted from evolutionary shifts in the regulation of developmental timing and events, that is, a phenomenon called heterochrony (Gould, 1977; Smith, 2001, 2003; Rice, 2002; Geuten & Coenen, 2013). As a fundamental aspect of all developmental processes, heterochrony may affect the evolution of development in plants by altering the timing of key developmental events, which enables the organism to better respond to changes in developmental and environmental cues (Keyte & Smith, 2014). Several studies have identified a particular set of endogenous machineries that control heterochrony (Moss, 2007; Huijser & Schmid, 2011). For example, in Arabidopsis, two components of the MEDIATOR CYCLIN DEPENDENT KINASE 8 (CDK8) module, Mediator complex subunit 12 encoded by *CENTER CITY* and Mediator complex subunit 13 encoded by *GRAND CENTRAL*, were found to be crucial for regulating the timing of radial pattern formation during early embryogenesis (Gillmor *et al*., 2010, 2014). Similarly, epigenetic regulation of gene expression through HISTONE 3 LYSINE 27 (H3K27) methylation was found to play an important role in affecting the timing of seed‐to‐seedling and vegetative‐to‐reproductive transitions in plants (Bastow *et al*., 2004; Bouyer *et al*., 2011; Crevillen & Dean, 2011). Nevertheless, these studies simply focused on particular pathways that cause heterochonic changes, and did not address the entire landscape of the genetic control of heterochrony in organ development.

More recently, Sun *et al*. (2014) have proposed a quantitative framework for characterizing the genetic architecture of heterochrony by mapping these so‐called heterochronic quantitative trait loci (*h* QTLs). This framework was founded on a dynamic mapping approach, called functional mapping, which integrates mathematical aspects of growth laws into a mapping setting to localize dynamic QTLs that govern the biological process of trait formation (Ma *et al*., 2002; Wu & Lin, 2006; He *et al*., 2010; Li & Wu, 2010; Zhao *et al*., 2012). By simultaneously modeling phenotypic measurements taken at a finite number of time‐points, functional mapping can provide biologically meaningful results that can be used to interpret the function of QTLs from a developmental perspective. Zeng *et al*. (2014) used functional mapping to detect and map four QTLs that control stem height and base diameter growth in juvenile seedlings of a coniferous tree, *Torreya grandis*. In many other studies, functional mapping has identified QTLs for key growth traits, such as whole‐plant biomass growth in soybean (*Glycine max*; Wu *et al*., 2011), plant height growth in rice (*Oryza sativa*; Zhao *et al*., 2004b), stemwood growth in Scots pine (*Pinus sylvestris;* Li *et al*., 2014), human height growth (Li *et al*., 2009) and mouse mass growth (Zhao *et al*., 2004a).

By further investigating whether and how these QTLs detected mediate the heterochronic pattern of trait growth, results from functional mapping can be upgraded not only to provide more useful information for designing breeding and management plans for agricultural crops, but also to predict the evolution of development (Rice, 2002, 2008).

The common bean has long served as one of the most important grain legume crops for human consumption and has also played a pivotal role in sustainable agriculture because of its ability to fix atmospheric nitrogen (Cichy *et al*., 2009). As the main source of photosynthetic products, leaf organs are particularly important for plant growth and production. Leaf morphological traits, which are well represented by leaf area and dry weight, are primary determinants of many physiological mechanisms, such as nutrient accumulation and water and energy exchange (Milla & Reich, 2007). In cereal crops, it was found that source leaves, particularly flag leaves, are associated with grain filling, 1000‐grain weight, panicle weight and many other yield‐related traits (Li *et al*., 1998; Quarrie *et al*., 2006). As a result of this, an understanding of the physiological roles of leaf area and dry weight is, therefore, of paramount relevance for improving plant growth. In the past few decades, a considerable body of studies has focused on the ecological function of leaf area and leaf dry weight across a range of environments (Yin *et al*., 1999; Milla & Reich, 2007), but knowledge of the underlying genetic control of these two traits is very limited. Byrne *et al*. (1997) used genetic mapping to detect two QTLs affecting leaf area in *Eucalyptus nitens*. Several QTLs were found to control the leaf area of single leaves located on different types of branch in *Populus* hybrids (Wu *et al*., 1997). In a genome‐wide association study (GWAS) of the maize (*Zea mays*) nested association mapping panel, Tian *et al*. (2011) characterized the polygenic inheritance of leaf size traits affected by many QTLs of small effects.

In this article, we describe the implementation of Sun *et al*.'s (2014) *h*QTL mapping model to identify and map specific *h* QTLs for leaf growth trajectories in a mapping population of the common bean (*Phaseolus vulgaris* L.), grown in two contrasting environments. The mapping population is composed of 177 recombinant inbred lines (RILs), derived from a Mesoamerican cultivar (Jamapa) and an Andean cultivar (Calima). This article reports the first study of its kind to attempt to elucidate the genetic architecture of the heterochrony of leaf area and dry weight growth trajectories and, more importantly, to characterize environment‐dependent changes of the effects of *h* QTLs on developmental timing. The *h*QTL mapping model identified two major *h* QTLs that pleiotropically determine leaf area and mass growth of the common bean in a growing season as well as each of these two traits expressed in two distinct environments. A trait‐ and environment‐specific *h*QTL was observed to operate under a particular environment. The identification of *h* QTLs may not only provide scientific guidance for marker‐assisted selection of economically important traits in the common bean, but will also help us to address fundamental questions about the genetic mechanisms of heterochrony that drive morphological diversification and evolution.

## Materials and Methods

### Mapping population

We obtained a mapping population composed of 177 recombinant inbred lines (RILs), derived from a Mesoamerican cultivar (Jamapa) and an Andean cultivar (Calima) of *Phaseolus vulgaris* L. These RILs and the two parents were genotyped for 513 molecular markers located on 11 linkage groups each covering a common bean chromosome (Bhakta *et al*., 2015).

### Experimental design and data collection

During 2011–2012, the mapping population (including both parents) was planted at two sites with contrasting temperature regimes in southwestern Colombia: Palmira and Popayan (Table 1). At each site, the field experiment was laid out in a randomized complete block row‐column design with three replicates (six for each parent) each with 35–50 plants per RIL. One plant from each replicate for each RIL was harvested weekly, starting with seedlings at approximately stage V0, which is when the primary leaves are unfurled, and ending with plants at stage R1, which is when the flowers are fully open and functional. In total, we conducted five weekly harvests. Because of differences in phenology, the first harvest was taken 15 and 18 days after planting at Palmira and Popayan, respectively. At each time of harvest, the first five leaves were measured independently for leaf area and leaf mass (dry weight), but only the first leaf was considered for this study because it is the one for which we had more complete data in a time series. Individual leaves were separated into petioles and laminas at node positions. Lamina area was measured with a Li‐Cor^®^ LI‐3100C area meter. A mix of petiole and lamina for each leaf was then dried in a forced‐air oven for 48 h at 55°C for dry weight determination. We took the mean of three replicates for each RIL at each time‐point for the subsequent statistical mapping analysis.

**Table 1:** General geographic and soil characteristics and relevant weather information for the two field sites in Colombia where the common bean (*Phaseolus vulgaris*) Jamapa × Calima recombinant inbred population was grown

	Site	
	Palmira (PAL)	Popayan (POP)
Latitude	03°29′N	02°25′N
Longitude	76°81′W	76°62′W
Altitude (m)	1000	1800
Soil type	Mollisol, aquichapludoll	Inceptisol, typic dystrandept
Growing season	11 Nov 2011 to Jan 2012	23 Mar 2012 to Jun 2012
Solar radiation (MJ m^−2^ d^−1^)	14.7	15.6
Temperature (min–max) (°C)	19.5–28.8	13.7–25.5
Day length (h)	11.8	12.1

Weather values represent averages calculated during the growing season at each site.

### Dissecting the growth curve

One of the most important equations for capturing age‐specific change in growth is the logistic curve (Niklas, 1994; West *et al*., 2001), which we used to describe leaf area and dry weight growth according to the following expression:

(Eqn 1) $$g\left(t\right)=\frac{a}{1+be^{-rt}}$$

(*g* (*t*), the trait value at time *t* ; *a*, the asymptotic value of *g* when *t *→ ∞; *b*, a parameter to position the curve on the time axis; *r*, the relative growth rate which determines the spread of the curve along the time axis.) Consequently, any specific growth characteristics described by the logistic growth function (Eqn 1) can be captured by estimating its parameters (*a*,* b* and *r*), and these in turn can be used to determine the coordinates of biologically important benchmarks along the growth trajectory.

There are three physiologically important benchmarks on the growth curve: the point of maximum acceleration, the inflection point, and the point of maximum deceleration, with coordinates denoted *P*_a_, *P*_I_ and *P*_d_, respectively. The inflection point *P*_I_ marks the point at which the relative growth rate reaches its maximum. The coordinates of *P*_I_ are obtained by calculating the second derivative of the growth equation, as

(Eqn 2) $$\left(t_{I},g_{I}\right)=\left(\frac{\log_{e}b}{r},\frac{a}{2}\right)$$

The growth curve is divided into two phases separated at *P*_I_, the exponential growth (from time *t* = 0 to *P*_I_) and the asymptotic growth (from *P*_I_ to infinite time).

The points *P*_a_ and *P*_d_ mark the timing of maximum acceleration and maximum deceleration of growth, which are the first and second inflection points of the growth rate curve, respectively. These two points partition the growth curve into three phases, the exponential growth phase (from time *t* = 0 to *P*_a_), the linear growth phase (from *P*_a_ to *P*_d_) and the ageing phase (from *P*_d_ onwards). By calculating the third derivative of the growth Eqn 1 with respect to time, the coordinates of *P*_a_ and *P*_d_ can be obtained as:

(Eqn 3) $$\left(t_{a},g_{a}\right)=\left(\frac{\log_{e}b\left(2-\sqrt{3}\right)}{r},\frac{a(3-\sqrt{3})}{6}\right)$$

(Eqn 4) $$\left(t_{d},g_{d}\right)=\left(\frac{\log_{e}b\left(2+\sqrt{3}\right)}{r},\frac{a(3+\sqrt{3})}{6}\right)$$

The duration of linear growth can be calculated as

(Eqn 5) $$\Delta T=t_{d}-t_{a}$$

According to Sun *et al*. (2014), four parameters, that is, the timing of the inflection point (Eqn 2), the timing of maximum acceleration of growth (Eqn 3), the timing of maximum deceleration of growth (Eqn 4), and the duration of linear growth (Eqn 5), are defined as the heterochronic parameters of growth processes. These heterochronic parameters are determined by the growth process, and define the growth trajectory of the organ under study (Smith, 2001; Rice, 2002).

### Modeling genetic variation in the growth curve through functional mapping

If specific QTLs exist to affect the dynamic change of a trait, the growth parameters that specify the change should be different among QTL genotypes. Functional mapping based on a mixture model‐based likelihood can be used to estimate QTL genotype‐specific parameters (Ma *et al*., 2002). For a particular trait, leaf area or leaf dry weight, this study contains two environments of the same mapping population which can be integrated within a joint framework of functional mapping, expressed as

(Eqn 6) $$L(\Phi |y,y^{\prime }=\prod_{i=1}^{n}\left[\omega_{1|i}f_{1}\left(y_{i},y_{i}^{\prime }\right)+\omega_{2|i}f_{2}\left(y_{i},y_{i}^{\prime }\right)\right]$$

where **Φ** represents the unknown parameters which include: the QTL position, the time‐dependent effects of different QTL genotypes, and the time‐dependent residual variances and correlations. (**y**_*i*_, **y**′_*i*_) = (*y*_*i*_(1), *y′*_*i*_(1), …, *y*_*i*_(5), *y′*_*i*_(5)) is the phenotypic vector of RIL *i* measured at five time‐points grown in Palmira (denoted by *y*) and Popayan (denoted by *y′*), respectively; ω_1|*i*_ and ω_2|*i*_ are the conditional probabilities of QTL genotypes *QQ* (coded by 1) and *qq* (coded by 2), respectively, given the marker genotype of RIL *i*; and *f*_1_(**y**_*i*_, **y**′_*i*_) and *f*_2_(**y**_*i*_, **y**′_*i*_) are a multivariate normal distribution with time‐dependent mean vector for genotype *QQ* and *qq*,

(Eqn 7) $$\begin{array}{cc} \left[u_{1},u_{1}^{\prime }\right]= & \left[\left(u_{1}\left(1\right),{u^{\prime }}_{1}\left(1\right)\right),\ldots ,\left(u_{1}\left(5\right),{u^{\prime }}_{1}\left(5\right)\right)\right]\text{.5 for}\;QQ\\ \left[u_{2},u_{2}^{\prime }\right]= & \left[\left(u_{2}\left(1\right),{u^{\prime }}_{2}\left(1\right)\right),\ldots ,\left(u_{2}\left(5\right),{u^{\prime }}_{2}\left(5\right)\right)\right]\text{.5 for}\;qq \end{array}$$

where *u* and *u′* denote the means for Palmira and Popayan, respectively, and (10 × 10)‐dimensional longitudinal covariance matrix, expressed as

(Eqn 8) $$\sum =\left(\begin{array}{cc} \sum_{1} & \sum_{12}\\ \sum_{21} & \sum_{2} \end{array}\right)$$

We assume that Σ_1_ and Σ_2_, the covariance matrices for Palmira and Popayan, respectively, have stationary structure, which can be modeled by the first‐order autoregressive (AR(1)) approach (Ma *et al*., 2002). The covariance matrix between Palmira and Popayan, Σ_12_ = Σ_21_, can be modeled as a separable structure (Mitchell *et al*., 2005). It should be noted that, because of their consistency at two sites, time‐points have been normalized as Eqns 1 to 5 in the joint model (Eqn 6).

Functional mapping models the time‐dependent genotypic values (Eqn 7) determined by a growth equation (Eqn 1). At two different sites, we will use a different set of growth parameters, that is, (*a*_11_, *b*_11_, *r*_11_) for genotype *QQ* and (*a*_12_, *b*_12_, *r*_12_) for genotype *qq* in Palmira and (*a*_21_, *b*_21_, *r*_21_) for genotype *QQ* and (*a*_22_, *b*_22_, *r*_22_) for genotype *qq* in Popayan. Functional mapping has been implemented with the EM algorithm to estimate these parameters.

Sun *et al*. (2014) proposed a procedure to test and estimate the effect of the *h* QTLs detected on heterochrony. Here, we list several key hypothesis tests of how an *h* QTL affects the heterochronic parameters and how it interacts with the environment:

1. the effect of *h* QTL on a heterochronic parameter within the environment;
2. the pleiotropic effect of *h* QTL on a heterochronic parameter of two environments;
3. the effect of the *h* QTL–environment interaction on a heterochronic parameter;
4. the plastic response of an *h* QTL genotype to environmental change.

In this study, we considered two different but allometrically related traits, leaf area and leaf mass. Functional mapping has been extended to map QTLs that contribute to the developmental correlation between different traits (Zhao *et al*., 2005). We used functional mapping to test how an *h* QTL affects pleiotropically a heterochronic parameter of the two traits.

## Results

### Leaf growth trajectories

**Figure 1:**
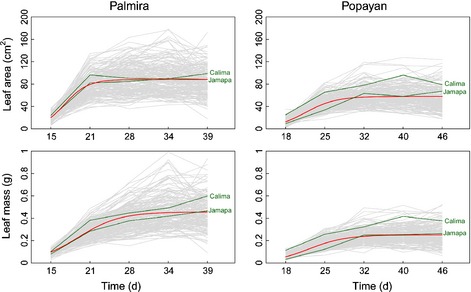
Growth trajectories for leaf area and mass from stage V0 to R (in days) for recombinant inbred lines (RILs) of the common bean (*Phaseolus vulgaris*) derived from a Mesoamerican cultivar (Jamapa) and an Andean cultivar (Calima), planted at two sites, Palmira and Popayan. The red line is the fit of the growth equation (Eqn 1) to the mean curves of all RILs, whereas the green lines are the observed data for the two parents.

By plotting leaf area and dry weight growth as a function of time at the two planting sites for each RIL and the parents (Fig. 1), we identified considerable genotypic variation in the time trajectories of both traits. In general, leaves were larger and heavier in Palmira than in Popayan, perhaps because the former was warmer than the latter. The patterns of growth at the two sites were similar from V1 (first trifoliate unfurled) to R1 (anthesis) stages, but started to diverge after R1. At Palmira, leaves of the parent Calima were slightly larger, but strikingly heavier than those of the parent Jamapa, whereas the parent Calima presented consistently larger values than the parent Jamapa for both leaf traits at Popayan.

Leaf area and mass growth trajectories were fitted by a logistic growth equation (Eqn 1), respectively, for each site. Results of fitness using a nonlinear least‐squares approach indicate that the mean growth of all RILs can be well described by the growth equation for both traits at both sites (*P *<* *0.001; Fig. 1). The shapes of fitted growth trajectories were markedly divergent for the same trait between different sites and also varied between the two traits at the same site. While the environment‐dependent difference may result from the response of plants to changing environment, the trait‐typical discrepancy implies the use of different developmental machineries for two allometrically related traits.

### Detection of growth QTLs and test of *h* QTLs

**Figure 2:**
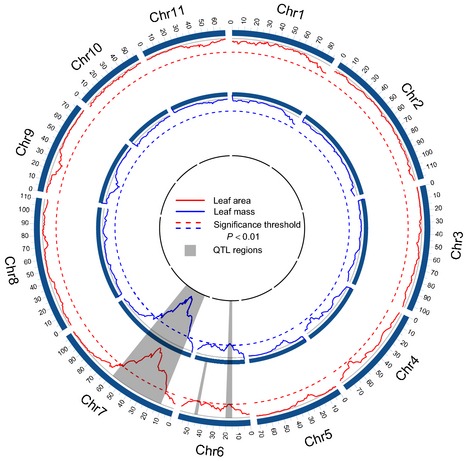
The profile of the log‐likelihood ratios that test the existence of quantitative trait loci (QTLs) for leaf area growth (red, outer circle) and leaf mass growth (blue, inner circle) of the common bean (*Phaseolus vulgaris*) grown at two sites across the 11 chromosomes. The genomic position corresponding to the peak of the curve is the maximum likelihood estimate of QTL locations. The map distances (in centiMorgans) between two markers are calculated using the Haldane mapping function. The broken lines indicate genome‐wide critical thresholds to declare the existence of a QTL obtained from 1000 permutation tests. The gray regions indicate the location at which significant QTLs reside. Two QTL regions for leaf area on chromosome 6 are separated by over 60 cM so that they are considered to harbor two independent QTLs. [Correction added after online publication 27 March 2015: figure and text altered to reflect updated nomenclature.]

The joint model of functional mapping (Eqn 2) by combining information from two sites was used to scan over 11 chromosomes for QTLs that control leaf growth. Three significant QTLs were identified for leaf growth trajectories, which we named *LeafG1*, located between markers DiM 7‐7 and DiM 7‐8 on chromosome 7, *LeafG2*, located between DiM 6‐15 and Bng088 on chromosome 6, and *LeafG3*, located between Bng183 and DiM 6‐25 on chromosome 6 (Fig. 2) [Correction added after online publication 27 March 2015: the chromosome number was updated in this sentence following a change in nomenclature]. *LeafG1* and *LeafG2* exert a pleiotropic effect on two different traits and also display an environmental pleiotropy on the same trait expressed in different environments. *LeafG3* is only responsible for leaf area growth, although it affects pleiotropically this trait at both sites.

**Figure 3:**
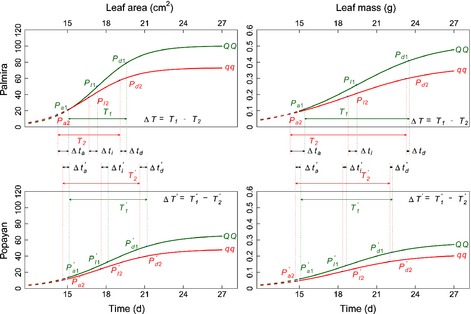
Genetic variation of developmental timing for leaf area and leaf mass caused by a heterochrony quantitative trait locus (*h*QTL) (*LeafG1*) detected between markers DiM 7‐7 and DiM 7‐8 on chromosome 7 in the common bean (*Phaseolus vulgaris*), grown in Palmira and Popayan [Correction added after online publication 27 March 2015: the marker names have been corrected.]. The three landmarks *P*_a_, *P*_I_ and *P*_d_ are the timing of maximum acceleration of growth, the timing of maximum rate of growth, and the timing of maximum deceleration of growth, respectively, and time interval *T* is the duration of linear growth. All these heterochronic parameters are subscribed by the QTL genotype, i.e. QQ (coded as 1) with alleles derived from the parent Calima and *qq* (coded as 2) with alleles from the parent Jamapa. The effects of the *h*QTL on heterochronic parameters are denoted as Δ*t*_a_, Δ*t*_I_, Δ*t*_d_ and Δ*T*, respectively, for the timing of maximum acceleration of growth, the timing of maximum rate of growth, the timing of maximum deceleration of growth, and the duration of linear growth. Popayan is distinguished from Palmira by a prime.

**Figure 4:**
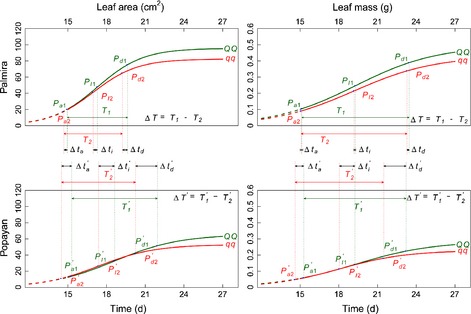
Genetic variation of developmental timing for leaf area and leaf mass caused by a heterochrony quantitative trait locus (*h*QTL) (*LeafG2*) detected between markers DiM 6‐15 and Bng088 on chromosome 6 in the common bean (*Phaseolus vulgaris*), grown in Palmira and Popayan [Correction added after online publication 27 March 2015: the marker names and location have been corrected.]. The effects of the *h*QTL on heterochronic parameters are denoted as Δ*t*_a_, Δ*t*_I_, Δ*t*_d_ and Δ*T* respectively, for the timing of maximum acceleration of growth, the timing of maximum rate of growth, the timing of maximum deceleration of growth, and the duration of linear growth. Popayan is distinguished from Palmira by a prime.

**Figure 5:**
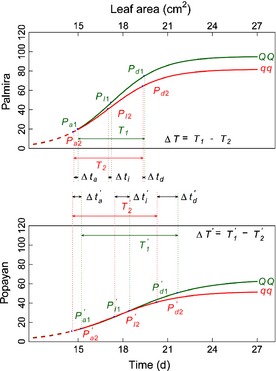
Genetic variation of developmental timing for leaf area and leaf mass caused by a heterochrony quantitative trait locus (*h*QTL) (*LeafG3*) detected between markers Bng183 and DiM 6‐25 on chromosome 6 in the common bean (*Phaseolus vulgaris*), grown in Palmira and Popayan [Correction added after online publication 27 March 2015: the marker names and location have been corrected.]. The effects of the *h*QTL on heterochronic parameters are denoted as Δ*t*_a_, Δ*t*_I_, Δ*t*_d_ and Δ*T*, respectively, for the timing of maximum acceleration of growth, the timing of maximum rate of growth, the timing of maximum deceleration of growth, and the duration of linear growth. Popayan is distinguished from Palmira by a prime.

Table 2 lists the estimates of the growth equation parameters (Eqn 1) for each of the two alleles from each detected QTL and at each of the two sites; also listed is the standard error of each estimate, which was obtained by a bootstrap resampling approach. It was seen that two genotypes at each QTL detected differed from each other in a manner depending on the type of leaf traits and the environment where the plants were grown (Figs 3, 4, 5), as a result of different temporal patterns of genetic effects triggered by a QTL. For the same trait, all QTLs were expressed more rapidly over time at Palmira than at Popayan. The alleles derived from the parent Calima (denoted as *QQ*) at all QTLs detected increased leaf area and mass growth at both sites, as compared with those from the parent Jamapa (denoted as *qq*). We used Sun *et al*.'s *h* QTL mapping model to test whether the QTLs detected govern heterochronic parameters of growth processes, that is, the timing of the inflection point, the timing of maximum acceleration, the timing of maximum deceleration, and the duration of linear growth. All the QTLs detected were found to be heterochronic in nature, and thus can be called *h* QTLs, because they are responsible for at least one heterochronic parameter for leaf trait growth at one or two sites (Table 3). *LeafG1* affected all four heterochronic parameters of leaf area growth when the plants were grown at Palmira, but it only controlled the timing of maximum acceleration for the same trait at Popayan. This environment‐dependent *h* QTL did not influence any heterochronic parameter for leaf mass growth at both sites. All this suggests that the expression of an *h* QTL can be sensitive to environmental change and may also vary depending on the type of trait.

**Table 2:** The maximum likelihood estimates (MLEs) of growth parameters (*a*,* b* and *r*) and standard errors of the estimates for three quantitative trait loci (QTLs) found to affect leaf area and leaf mass growth in the common bean (*Phaseolus vulgaris*) grown at two different sites, Palmira and Popayan

		Leaf area		Leaf mass	
		Palmira	Popayan	Palmira	Popayan
*LeafG1*		Position: DiM 7‐7 – DiM 7‐8			
*QQ*	*a*	100.23 ± 1.04	66.08 ± 0.84	0.52 ± 0.006	0.28 ± 0.004
	*b*	132.71 ± 20.27	54.13 ± 8.26	30.00 ± 3.17	35.78 ± 3.66
	*r*	3.51 ± 0.11	2.61 ± 0.08	1.95 ± 0.06	2.22 ± 0.06
*qq*	*a*	73.11 ± 1.10	48.47 ± 0.72	0.38 ± 0.005	0.21 ± 0.002
	*b*	66.31 ± 20.47	44.70 ± 7.91	17.47 ± 3.54	29.10 ± 4.16
	*r*	3.28 ± 0.23	2.64 ± 0.11	1.75 ± 0.09	2.16 ± 0.08
*LeafG2*		Position: DiM 6‐15 – Bng088			
*QQ*	*a*	95.54 ± 1.18	65.35 ± 0.97	0.49 ± 0.007	0.28 ± 0.004
	*b*	107.13 ± 11.66	45.52 ± 3.09	26.01 ± 1.48	30.18 ± 1.32
	*r*	3.38 ± 0.08	2.38 ± 0.05	1.90 ± 0.04	1.99 ± 0.03
*qq*	*a*	82.33 ± 1.03	52.88 ± 0.67	0.43 ± 0.006	0.23 ± 0.003
	*b*	101.31 ± 10.57	46.80 ± 3.69	26.33 ± 1.12	31.99 ± 1.84
	*r*	3.48 ± 0.10	2.75 ± 0.06	1.93 ± 0.03	2.30 ± 0.04
*LeafG3*		Position: Bng183 – DiM 6‐25			
*QQ*	*a*	95.14 ± 1.36	64.26 ± 1.11		
	*b*	129.06 ± 10.27	47.07 ± 2.94		
	*r*	3.54 ± 0.08	2.44 ± 0.04		
*qq*	*a*	81.86 ± 1.41	51.99 ± 0.67		
	*b*	94.78 ± 9.13	50.56 ± 4.36		
	*r*	3.40 ± 0.09	2.78 ± 0.06		

[Correction added after online publication 27 March 2015: the marker names have been corrected.]

**Table 3:** The genetic and environment effects of three quantitative trait loci (QTLs) detected on four heterochronic parameters of leaf area and leaf mass growth in the common bean (*Phaseolus vulgaris*)

		Leaf area				Leaf mass			
		Palmira		Popayan		Palmira		Popayan	
		*QQ*	*qq*	*QQ*	*qq*	*QQ*	*qq*	*QQ*	*qq*
*LeafG1*									
*t*_I_	Value	1.39	1.28	1.53	1.44	1.74	1.63	1.61	1.56
	Genetic effect	0.11**		0.09^ns^		0.11^ns^		0.05^ns^	
	Environmental effect	−0.14**			−0.16**	0.13**			0.07^ns^
*t*_a_	Value	1.02	0.88	1.02	0.94	1.07	0.88	1.02	0.95
	Genetic effect	0.14*		0.08**		0.19^ns^		0.07^ns^	
	Environmental effect	0.00^ns^			−0.06^ns^	0.05^ns^			−0.07^ns^
*t*_d_	Value	1.77	1.68	2.03	1.94	2.42	2.39	2.20	2.17
	Genetic effect	0.09**		0.09^ns^		0.03^ns^		0.03^ns^	
	Environmental effect	−0.26**			−0.26**	0.22**			0.22*
*∆T*	Value	0.75	0.80	1.01	1.00	1.35	1.51	1.18	1.22
	Genetic effect	−0.05**		0.01^ns^		−0.16^ns^		−0.04^ns^	
	Environmental effect	−0.26**			−0.20**	0.17*			0.29*
*LeafG2*									
*t*_I_	Value	1.38	1.33	1.60	1.40	1.71	1.70	1.71	1.51
	Genetic effect	0.05*		0.20**		0.01^ns^		0.20*	
	Environmental effect	−0.22**			−0.07*	0.00^ns^			0.19**
*t*_a_	Value	0.99	0.95	1.05	0.92	1.02	1.01	1.05	0.93
	Genetic effect	0.04^ns^		0.13^ns^		0.01^ns^		0.12^ns^	
	Environmental effect	−0.06^ns^			0.03^ns^	−0.03^ns^			0.08^ns^
*t*_d_	Value	1.77	1.71	2.16	1.88	2.41	2.38	2.37	2.08
	Genetic effect	0.06^ns^		0.28**		0.03^ns^		0.29**	
	Environmental effect	−0.45**			−0.17*	0.04^ns^			0.3**
*∆T*	Value	0.78	0.76	1.11	0.96	1.39	1.37	1.32	1.15
	Genetic effect	0.02^ns^		0.15*		0.02^ns^		0.17*	
	Environmental effect	−0.33**			−0.2^ns^	0.07^ns^			0.22*
*LeafG3*									
*t*_I_	Value	1.37	1.34	1.58	1.41				
	Genetic effect	0.03^ns^		0.17*					
	Environmental effect	−0.21**			−0.07^ns^				
*t*_a_	Value	1.00	0.95	1.04	0.94				
	Genetic effect	0.05^ns^		0.10*					
	Environmental effect	−0.04^ns^			0.01^ns^				
*t*_d_	Value	1.74	1.73	2.11	1.88				
	Genetic effect	0.01^ns^		0.23**					
	Environmental effect	−0.37**			−0.15^ns^				
*∆T*	Value	0.74	0.78	1.07	0.94				
	Genetic effect	−0.04^ns^		0.13*					
	Environmental effect	−0.33**			−0.16^ns^				

The genetic effect is defined as the difference in genotypic values between two alternative QTL genotypes at the same site (given between genotypes *QQ* and *qq*), whereas the environmental effect is defined as the difference in the same QTL genotypes between two sites (given between genotypes *QQ* and *qq*, respectively).

Significance: *, 0.05, **, 0.01; ^ns^, nonsignificant. *t*_I_, the timing of the inflection point; *t*_a_, the timing of maximum acceleration; *t*_d_, the timing of maximum deceleration; *∆T* = *t*_d_ − *t*_a_, the duration of linear growth.

*LeafG2* showed a different pattern of environment‐dependent genetic effects on heterochrony (Fig. 4; Table 3). Heterochronic parameters for both leaf traits were more likely to be affected by this *h* QTL when the plants were grown at Popayan than at Palmira. *LeafG2* had no effect on the heterochrony of leaf mass growth at Palmira. As a QTL specifically for leaf area growth, the heterochronic nature of *LeafG3* was also environment dependent. It affected the timing of the inflection point, the timing of maximum acceleration and the timing of maximum deceleration for leaf mass growth only when the plants were grown at Popayan (Fig. 5; Table 3).

### Environmental impact on the allelic expression of *h* QTL

**Figure 6:**
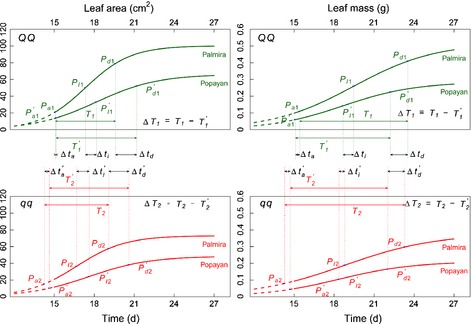
Plastic response of genotypes *QQ* and *qq* at *LeafG1*, with alleles derived from common bean (*Phaseolus vulgaris*) parents Calima and Jamapa, respectively, to environmental changes (Palmira and Popayan) for leaf area and leaf mass growth. The effects of the heterochrony quantitative trait locus (*h*QTL) on heterochronic parameters are denoted as Δ*t*_*a*_, Δ*t*_*I*_, Δ*t*_*d*_ and Δ*T*, respectively, for the timing of maximum acceleration of growth, the timing of maximum rate of growth, the timing of maximum deceleration of growth, and the duration of linear growth. Popayan is distinguished from Palmira by a prime.

We also tested how the environment affects the expression of *h* QTL genotypes by using Sun *et al*.'s (2014) model. The two *LeafG1* alleles, derived from Calima and Jamapa, displayed similar environmental responses for all heterochronic parameters, except for the timing of maximum acceleration (Fig. 6; Table 3). Compared with cooler Popayan, warmer Palmira could accelerate the occurrence of the inflection point and maximum deceleration and shorten the duration of linear growth for leaf area growth in *LeafG1* genotypes. However, the inverse pattern was found for leaf mass growth. In some cases, *LeafG1* displayed a significant effect of the QTL–environment interaction on heterochrony for two leaf traits (Table 3).

**Figure 7:**
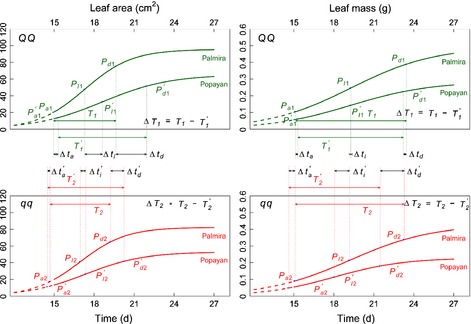
Plastic response of genotypes *QQ* and *qq* at *LeafG2*, with alleles derived from common bean (*Phaseolus vulgaris*) parents Calima and Jamapa, respectively, to environmental changes (Palmira and Popayan) for leaf area and leaf mass growth. The effects of the heterochrony quantitative trait locus (*h*QTL) on heterochronic parameters are denoted as Δ*t*_*a*_, Δ*t*_*I*_, Δ*t*_*d*_ and Δ*T*, respectively, for the timing of maximum acceleration of growth, the timing of maximum rate of growth, the timing of maximum deceleration of growth, and the duration of linear growth. Popayan is distinguished from Palmira by a prime.

Like *LeafG1*, genotypes of *LeafG2* were not affected for the timing of maximum acceleration by the environment (Fig. 7). Also, this *h* QTL's genotypes were accelerated in the timing of development by warmer Palmira which shortened the duration of linear growth for leaf area. It is interesting to see that the environment did not affect the heterochrony of leaf mass growth for the genotype *QQ* at *LeafG2* with alleles from Calima, but did so for genotype *qq* with alleles from Jamapa. QTL–environment interactions were quite common in heterochrony for *LeafG2*.

**Figure 8:**
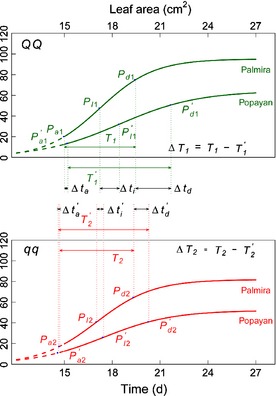
Plastic response of genotypes *QQ* and *qq* at *LeafG3*, with alleles derived from common bean (*Phaseolus vulgaris*) parents Calima and Jamapa, respectively, to environmental changes (Palmira and Popayan) for leaf area and leaf mass growth. The effects of the heterochrony quantitative trait locus (*h*QTL) on heterochronic parameters are denoted as Δ*t*_*a*_, Δ*t*_*I*_, Δ*t*_*d*_ and Δ*T*, respectively, for the timing of maximum acceleration of growth, the timing of maximum rate of growth, the timing of maximum deceleration of growth, and the duration of linear growth. Popayan is distinguished from Palmira by a prime.

At *LeafG3*, Jamapa allelic genotype *qq* was not responsive to the environment in four heterochronic parameters for leaf area growth, but genotype *QQ* composed of Calima alleles displayed a significant difference in most parameters between the two environments (Fig. 8; Table 3). The pattern of environmental influence on leaf area growth for genotype *QQ* was similar to those of the other two *h* QTLs. Also, QTL–environment interactions for *LeafG3* were observed.

## Discussion

There is a rich body of evidence indicating that leaf area is positively correlated with growth and productivity in annual crops (Li *et al*., 1998; Vos *et al*., 2005; Quarrie *et al*., 2006) and perennial trees (Wu & Stettler, 1994; Byrne *et al*., 1997). The reason leaf area and leaf mass play a pivotal role in plant growth and productivity is that the leaf is the organ where the majority of light interception and carbon fixation takes place. Genetic mapping has been used to identify specific QTLs that control leaf area and its relationship to grain yield in rice (Li *et al*., 1998) and barley (*Hordeum vulgare*; Yin *et al*., 1999) and stemwood growth in poplar (Wu *et al*., 1997) and eucalyptus (Byrne *et al*., 1997). All these findings have provided important information to enhance the molecular breeding of yield traits through marker‐assisted selection of leaf area.

While many studies in the literature have focused on the identification of QTLs for leaf traits measured at a single time‐point, the current study in common bean attempted to map leaf QTLs from a dynamic perspective, producing several novel findings. First, we used functional mapping to detect QTLs that control leaf growth traits and to characterize their temporal pattern of gene action. The QTLs we detected increased leaf area and leaf mass. Second, this study revealed the intriguing complexity of some QTL‐by‐environment interactions, a phenomenon of widespread occurrence in biological systems (El‐Soda *et al*., 2014). Although the three QTLs were found to affect leaf area expansion at both sites, the environment dictated what dynamic aspects of the QTL would be effective. For instance, the *LeafG1* Calima allele had a significant effect on the timing of the inflection point, maximum acceleration, maximum deceleration, and duration of linear growth in Palmira, but it only had a significant effect on the timing of maximum acceleration in Popayan. QTL‐by‐environment interactions of different complexities were also detected at the other QTLs. Third, we investigated two leaf traits, leaf area and leaf mass. Functional mapping allowed us to obtain a picture of pleiotropic control over these two allometrically related but developmentally different traits. *LeafG1* and *LeafG2* are strong pleiotropic QTLs that affect both leaf area and leaf mass, whereas *LeafG3* only affects leaf area growth. All QTLs are environmentally pleiotropic by triggering their effect on the same leaf traits expressed in different environments. Environmental pleiotropy may play an important role in the production of phenotypic plasticity and in adaptive evolution (Szamecz *et al*., 2014). Finally, the most important and significant contribution of this study is the use of a modified model of functional mapping (Sun *et al*., 2014) to characterize the dynamic mechanism of leaf growth and development by dissecting it into its heterochronic components.

Our study is the first of its kind to map specific QTLs for heterochrony, designated *h* QTL by Sun *et al*. (2014). We found three leaf growth *h* QTLs in the common bean with a characteristic heterochronic nature, which was manifested through significant alterations in the timing of several key growth and developmental events. These included the timing of maximum growth rate, the timing of maximum acceleration, the timing of maximum deceleration, and the duration of linear growth. Furthermore, our analysis revealed diverse and complex genotype‐by‐environment interactions for each of the *h* QTLs. The timing of the four heterochronic characters controlled by each of the *h* QTLs was not affected uniformly by the environmental sites. These results showed that the *h* QTL functional mapping model can reveal more intricate details of environmental responses of dynamic traits such as leaf growth, or any other similar trait. These results also show the dynamic complexity of growth and developmental mechanisms plant can use for adaptation to different environments (Geuten & Coenen, 2013). Heterochrony genes control precisely timed switches for developmental transitions, and provide in this way a time dimension to developmental regulation (Moss, 2007; Geuten & Coenen, 2013). Some heterochrony genes may possess homologs in other species. Thus, determining the identity of *h* QTLs detected in this study may have applications beyond the common bean.

Heterochrony is regarded as an exceedingly important process by which development can be modified to engender evolutionary changes (Smith, 2003; Keyte & Smith, 2014). In fact, heterochrony has been studied within explicit phylogenetic contexts as a concept linking evolution and development for over a century. The past decade has seen a tremendous increase in interest in the study of heterochrony, from the whole organism to the organ, cell, molecule, and gene level (Rice, 2002; Carleton *et al*., 2008; Wu *et al*., 2009; Moda *et al*., 2013). Our study focusing on mapping, under different environments, heterochrony genes controlling leaf growth and development provides fuel for efforts aiming to link fundamental questions of morphological diversity and phenotypic evolution in plants with the mechanistic pathways underlying these questions.
